# The anesthetic management of a patient undergoing simultaneous open abdominal aortic aneurysm repair and coronary artery bypass grafting: A case report

**DOI:** 10.1097/MD.0000000000031485

**Published:** 2022-11-11

**Authors:** Xu’an Wang, Jun Ma, Xiuhua Dong, Yang Bai, Dongni Zhang

**Affiliations:** a Department of Anesthesiology, Beijing Anzhen Hospital, Capital Medical University, Beijing, China.

**Keywords:** coronary artery bypass grafting, open abdominal aortic aneurysm repair, simultaneous surgery

## Abstract

Surgery for abdominal aortic aneurysm (AAA) and concomitant severe coronary artery disease (CAD) is usually managed in a staged procedure. The anesthesia for concurrent surgery is rare and complex. In this report, we present an unusual case of undergoing simultaneous open abdominal aortic aneurysm (AAA) repair and coronary artery bypass grafting (CABG).

**Patient concerns:**

A 70-year-old male AAA patient with concurrent triple-vessel CAD underwent a simultaneous surgery.

**Diagnosis:**

The patient underwent computed tomography angiography (CTA) and coronary angiography. He was diagnosed with AAA and triple-vessel CAD.

**Interventions:**

The patient underwent simultaneous surgery.

**Outcomes:**

The patient underwent anesthesia and surgery smoothly and was discharged on the 13th postoperative day.

**Lessons:**

The anesthetic management of simultaneous open abdominal aortic aneurysm repair and coronary artery bypass grafting is rare and complicated. Reasonable operation and anesthesia protocols, close monitoring and management of hemodynamic changes, and appropriate cell salvage and hemostasis measures are of great significance to increase perioperative safety and reduce the risk of postoperative complications.

## 1. Introduction

Abdominal aortic aneurysm (AAA) is an asymptomatic but potentially life-threatening condition due to the risk of rupturing. Anesthetic management is more complex when patients with AAA have concurrent triple-vessel coronary artery disease (CAD) since an AAA needs different or even opposite anesthetic strategies than CAD. Here the authors present a case of a patient undergoing simultaneous open AAA repair and coronary artery bypass grafting (CABG) and describe the anesthetic considerations.

## 2. Case report

**Figure 1.**
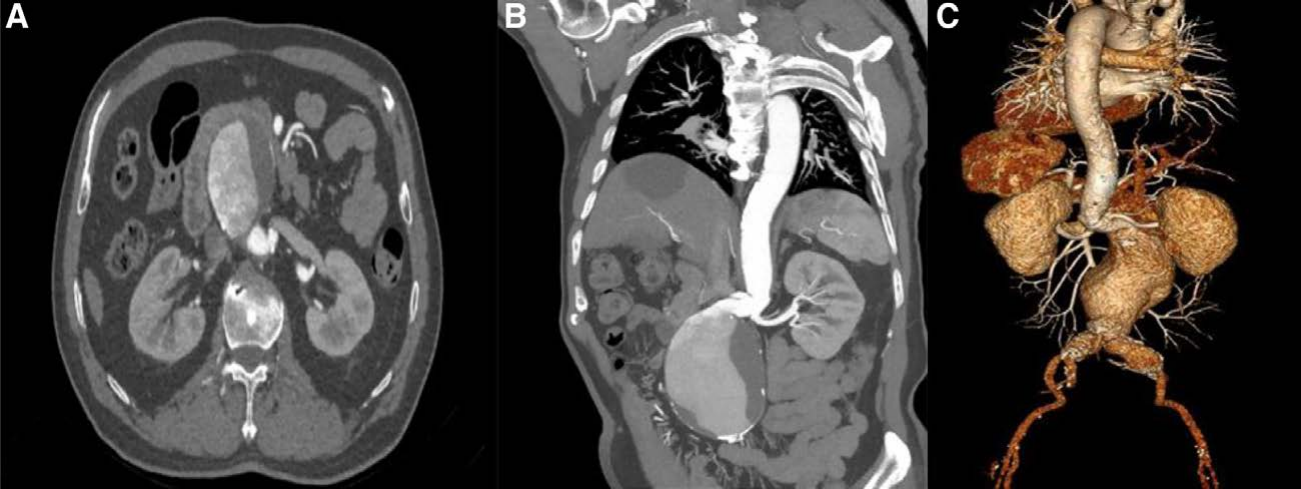
Preoperative computed tomography angiography revealed the infrarenal abdominal aortic aneurysm. A: Axial image of the abdominal cavity; B: Sagittal image; C: 3D reconstruction.

**Figure 2.**
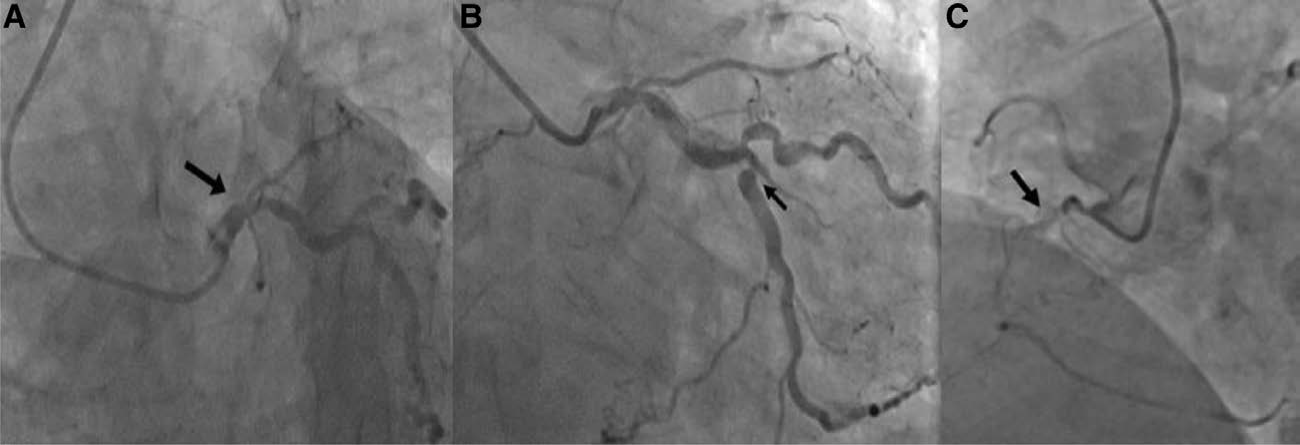
Preoperative coronary angiography revealed 3-vessel disease. A: Occlusion in left anterior descending artery (arrow); B: Severe stenosis in obtuse marginal branch (arrow); C: Occlusion in right coronary artery (arrow).

A 70-year-old male patient (weight, 73 kg; height, 173 cm) presented to the authors’ institution for a pulsatile abdominal mass. He had a 5-year medical history of coronary artery disease presenting as chest tightness after physical activity. Computed tomography angiography revealed aneurysmal dilatation of the abdominal aorta 4.5 mm below the left renal artery, with an internal diameter of approximately 87.6 mm and an involved length of approximately 135.4 mm (Fig. 1). Coronary angiography revealed occlusion of left anterior descending artery and right coronary artery and severe stenosis of the obtuse marginal branches (OM) (Fig. 2). The blood pressure (BP) of this patient was 116/66 mm Hg in the right upper extremity, 112/67 mm Hg in the right lower extremity, 107/65 mm Hg in the left upper extremity and 121/66 mm Hg in the left lower extremity. His treatment plan was discussed at a multidisciplinary meeting, where open abdominal aortic aneurysm repair and coronary artery bypass grafting were planned concurrently under cardiopulmonary bypass (CPB).

In the operating room, a small dose of midazolam was given for sedation after verifying the electrocardiogram, BP and oxygen saturation. Arterial catheters were then inserted into the right radial artery and right dorsal pedis artery with local anesthesia to monitor upper and lower extremity BP simultaneously. General anesthesia was induced with lidocaine, etomidate, rocuronium and sufentanil, followed by endotracheal intubation, and maintained with propofol, dexmedetomidine, sufentanil and rocuronium. A central venous catheter and a Swan-Ganz catheter were inserted into the right internal jugular vein under ultrasound guidance for the administration of vasoactive and positive inotropic medication and the measurement of the central venous pressure (CVP), pulmonary artery pressure (PAP) and cardiac index (CI). The patient’s BP in the right upper extremity before surgical incision was 121/60 mm Hg, his CVP was 4 mm Hg, his PAP was 21/9 mm Hg, and the CI was 2.5 L/min/m^2^.

To maintain arterial BP within the normal range before CPB, appropriate amounts of crystalloid solutions and vasoactive drugs (such as noradrenaline, methoxamine, and nicardipine) were administered. After heparinization, the patient first underwent CABG via CPB. A single saphenous vein graft was anastomosed to the left anterior descending artery. Then a sequential surgical technique with a saphenous vein was used in grafts of the first OM (OM_1_), second OM (OM_2_), and posterior descending artery. After the ascending aorta was unclamped and the heartbeat returned spontaneously, the coronary bypass flow velocity was adequate as measured by a flow meter. Subsequently, the abdominal aorta was cross-clamped above the left renal artery, the infrarenal aorta was transected, an interposition graft was sewn into place, and the abdominal aorta was unclamped. This step took approximately 10 minutes. Blood flow was restored in both lower extremities after the bilateral iliac arteries were sequentially anastomosed. Blood lost during the surgical procedure was collected into the CPB and a cell salvage system. After grafting and weaning from CPB, protamine was administered for heparin reversal. Subsequently, 1 g of tranexamic acid, 2 g of fibrinogen and 800 U of prothrombin complex concentrates were infused into the patient slowly to reduce bleeding. Dopamine and nitroglycerin were pumped to improve myocardial contractility and hemodynamic stability, and potassium and insulin were supplemented according to blood gas analysis. After hemostasis and flushing, the thoracic and abdominal cavities were closed and the patient was transferred to the intensive care unit. When leaving the operating room, the patient’s BP in the right upper extremity was 102/66 mm Hg, his CVP was 6 mm Hg, his PAP was 17/9 mm Hg, and the CI was 2.1L/min/m^2^.

**Figure 3.**
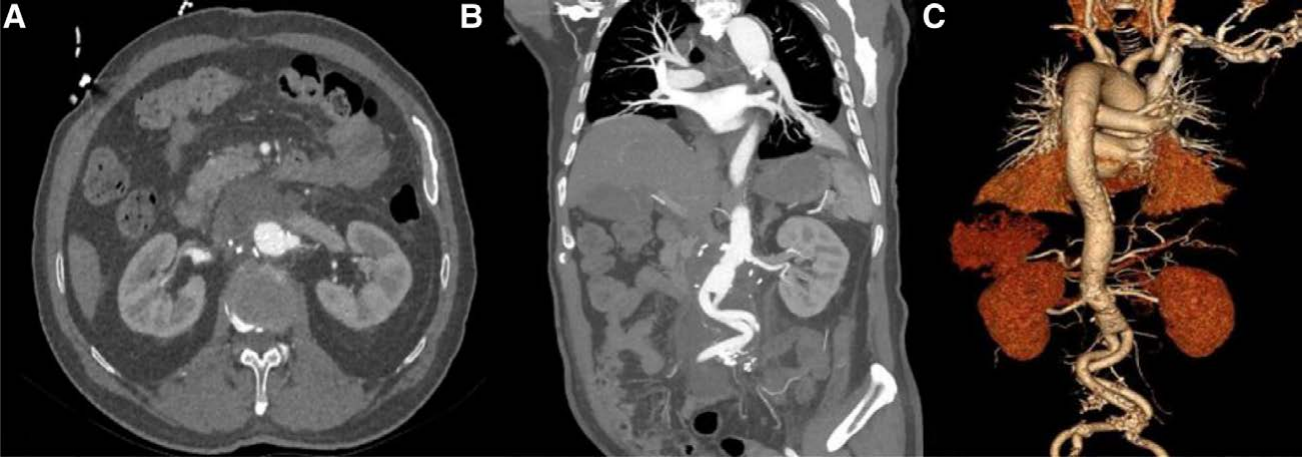
Postoperative computed tomography angiogram revealed an unobstructed vascular graft of abdominal aorta. A: Axial image of the abdominal cavity; B: Sagittal image; C: 3D reconstruction.

The operation lasted 8 hours, and the total volume of blood lost was 1200 mL. The total volume of transfusion was 7060 mL, consisting of 6360 mL of crystalloids and 4 units of packed red blood cells. The urine output during the operation was 4500 mL. The endotracheal tube was successfully removed on the first postoperative day, and computed tomography angiography revealed an unobstructed vascular graft of abdominal aorta on the 7th postoperative day (Fig. 3). The patient was discharged from the hospital on the 13th postoperative day.

## 3. Discussion

The development of AAA shares common risk factors with the development of CAD, including advanced age, smoking history, hypercholesterolemia, hypertension and obesity.^[1,2]^ The prevalence of CAD in patients with AAA has been estimated to be as high as 50%.^[3]^ The risks of perioperative mortality, myocardial ischemia, and adverse long-term outcomes are significantly increased in patients undergoing AAA repair with comorbid CAD.^[4]^ In patients with severe CAD and peripheral vascular disease, the usual treatment is first myocardial revascularization or percutaneous coronary intervention followed by AAA repair (staging surgery).^[5]^ It is rare and challenging to perform concurrent open surgery with AAA repair and CABG. In our patient, endovascular stenting was not considered an option because of the close relationship of the aortic aneurysm to the left renal artery. Out of concern for the risk of perioperative myocardial infarction or rupture of the large diameter AAA in this patient, a combined and open procedure was conducted.

Anesthetic management of AAA surgery requires the prevention of excessive rise in BP to rupture the aneurysm. Conversely, patients undergoing CABG need to avoid excessive BP drop to maintain adequate coronary perfusion pressure. In this complex setting, close monitoring and maintenance of cardiac performance are necessary. The placement of a pulmonary artery catheter (Swan–Ganz catheter) was appropriate in this patient for the real-time assessment of left ventricular function by measuring the pulmonary artery wedge pressure, PAP and CI. Transesophageal echocardiography is also invaluable for assessing changes in cardiac function and guiding intraoperative management. Abdominal aortic cross-clamping can cause a sudden increase in cardiac afterload and filling pressure. Excessive elevation of the PCWP and PAP may indicate cardiac dysfunction, especially in patients with severe CAD. In contrast, hypotension due to a sudden decrease in peripheral vascular resistance after unclamping can reduce coronary perfusion and even lead to myocardial ischemia. Close monitoring, careful fluid management and the appropriate use of vasoactive drugs are vital at these critical points.

Drastic alterations in cardiac afterload during aortic cross-clamping and unclamping in open AAA repair might be unacceptable in patients with severe CAD. Therefore, CABG should be performed first to relieve symptoms of myocardial ischemia and maximally prevent acute heart failure in the simultaneous surgery. Besides, CPB could protect cardiac function from dramatic hemodynamic changes during this concurrent operation. However, the timing of weaning from CPB remains controversial. Some studies advocate that CPB should be continued for mechanical cardiac assistance until aortic surgery is fully completed, even after myocardial revascularization.^[4,6]^ Some scholars argue that the slowly recovering ventricular function due to myocardial ischemia and reperfusion injury during CABG is intolerable due to the rapid changes in cardiac afterload and filling pressure during aortic cross-clamping and unclamping. However, other scholars are more concerned about the poor prognosis caused by excessive bleeding because of the prolonged CPB time.^[7]^ In this patient, extracorporeal support was continued during myocardial revascularization and aortic repair, and uncontrollable bleeding was not observed during the entire perioperative period.

Nitroglycerin is known to promote the vasodilation of capacitance veins and conductance arteries by releasing nitric oxide. Some studies have shown that an infusion of nitroglycerin initiated at rewarming by CPB can improve metabolic homeostasis without increasing the risk of stroke and requirement of renal replacement therapy.^[8,9]^ In addition, nitroglycerin could also increase coronary blood flow by dilating the epicardial coronary arteries and reducing the ventricular filling pressure, wall tension, and myocardial oxygen demand via vasodilation.^[10]^ In this patient, nitroglycerin was continuously infused from rewarming by CPB to the end of the operation to correct microcirculatory dysregulation and improve tissue perfusion.

Combined epidural-general anesthesia during open AAA repair may be associated with reducing the usage of narcotic drugs, providing sufficient abdominal relaxation and contributing to postoperative analgesia. Previous research^[11]^ has shown that combined epidural-general anesthesia can improve 30-day survival by improving organ perfusion and reducing postoperative complications compared with general anesthesia alone. However, combined epidural-general anesthesia has not been studied for simultaneous open AAA repair and CABG surgery. Considering the potential adverse effects of hemodynamic changes caused by an epidural block, general anesthesia alone was selected for this patient.

Renal dysfunction is a common complication after abdominal aortic aneurysm surgery and is associated with lower rates of survival and higher rates of long-term cardiovascular events.^[12]^ The juxtarenal abdominal aneurysm (one involving or near the renal artery) necessitates aortic cross-clamping above at least 1 renal artery, resulting in varying degrees of renal ischemia during repair. Several studies suggest that preoperative renal function, the aortic clamp position and the renal ischemia time are closely associated with adverse postoperative renal function.^[13,14]^ They subsequently pointed out that cold renal perfusion is useful for decreasing the rate of acute kidney injury in patients with a clamping time of more than 25 minutes. Cold renal perfusion was not employed in our patient because of the short clamping time, and there was no renal dysfunction during the perioperative period.

AAA repair is also associated with predictable blood loss due to surgical factors and acute coagulopathy. A high volume of intraoperative blood loss and the resulting blood transfusion are associated with increased complications and mortality.^[15]^ Intraoperative cell salvage could assist in blood conservation and reduce the number of transfused units, which has been recommended in operations with a substantial volume of anticipated blood loss.^[16]^ Tranexamic acid could exert an antifibrinolytic effect by reversibly blocking plasminogen binding on the surface of fibrin. A recent randomized clinical trial found a statistically significant reduction in postoperative bleeding with tranexamic acid in open aortic aneurysm surgery.^[17]^ In addition, fibrinogen and prothrombin complex concentrates, provided as substitutes for cryoprecipitate and fresh frozen plasma, could also be considered to treat post-CPB bleeding and reduce blood transfusion in cardiovascular surgery.^[16]^

In conclusion, the anesthetic management of simultaneous open abdominal aortic aneurysm repair and coronary artery bypass grafting is rare and complicated that requires additional research. Reasonable operation and anesthesia protocols, close monitoring and management of hemodynamic changes, and appropriate cell salvage and hemostasis measures are of great significance to increase perioperative safety and reduce the risk of postoperative complications.

## Author contributions

**Conceptualization:** Jun Ma, Xiuhua Dong.

**Data curation:** Xu’an Wang, Yang Bai.

**Investigation:** Xu’an Wang, Dongni Zhang.

**Supervision:** Jun Ma, Xiuhua Dong.

**Writing – original draft:** Xu’an Wang.

**Writing – review & editing:** Xiuhua Dong, Jun Ma.
